# Modeling Upper Limb Rehabilitation-Induced Recovery After Stroke: The Role of Attention as a Clinical Confounder

**DOI:** 10.1093/ptj/pzae148

**Published:** 2024-10-10

**Authors:** Silvia Salvalaggio, Simone Gambazza, Martina Andò, Ilaria Parrotta, Francesca Burgio, Laura Danesin, Pierpaolo Busan, Sara Zago, Dante Mantini, Daniela D’Imperio, Marco Zorzi, Nicola Filippini, Andrea Turolla

**Affiliations:** IRCCS San Camillo Hospital, Venice, Italy; Healthcare Professions Department, Fondazione IRCCS Ca’ Granda Ospedale Maggiore Policlinico, Milan, Italy; Department of Clinical Sciences and Community Health, Laboratory of Medical Statistics, Biometry and Epidemiology “G.A. Maccacaro”, University of Milan, Milan, Italy; Fondazione Don Gnocchi, “Centro S.M. della Provvidenza”, Rome, Italy; IRCCS San Camillo Hospital, Venice, Italy; Movement Control and Neuroplasticity Research Group, KU Leuven, Belgium; IRCCS San Camillo Hospital, Venice, Italy; IRCCS San Camillo Hospital, Venice, Italy; IRCCS San Camillo Hospital, Venice, Italy; IRCCS San Camillo Hospital, Venice, Italy; Movement Control and Neuroplasticity Research Group, KU Leuven, Belgium; IRCCS San Camillo Hospital, Venice, Italy; IRCCS San Camillo Hospital, Venice, Italy; Department of General Psychology and Padova Neuroscience Center, University of Padova, Padova, Italy; IRCCS San Camillo Hospital, Venice, Italy; Department of Biomedical and Neuromotor Sciences – DIBINEM, Alma Mater Studiorum Università di Bologna, Bologna, Italy; Unit of Occupational Medicine, IRCCS Azienda Ospedaliero-Universitaria di Bologna, Bologna, Italy

**Keywords:** Attention, Motor Function, Neuroimaging, Neurophysiology, Prediction, Rehabilitation, Statistical Models, Stroke

## Abstract

**Objective:**

People who have survived stroke may have motor and cognitive impairments. High dose of motor rehabilitation was found to provide clinically relevant improvement to upper limb (UL) motor function. Besides, mounting evidence suggests that clinical, neural, and neurophysiological features are associated with spontaneous recovery. However, the association between these features and rehabilitation-induced, rather than spontaneous, recovery has never been fully investigated. The objective was to explore the association between rehabilitation dose and UL motor outcome after stroke, as well as to identify which variables can be considered potential candidate predictors of motor recovery.

**Methods:**

People who survived stroke were assessed before and after a period of rehabilitation using motor, cognitive, neuroanatomical, and neurophysiological measures. We investigated the association between dose of rehabilitation and UL response (ie, Fugl-Meyer Assessment for upper extremity [FMA-UE]), using ordinary least squares regression as the primary analysis. To obtain unbiased estimates, adjusting covariates were selected using a directed acyclic graph.

**Results:**

Baseline FMA-UE was the only factor associated with motor recovery (b = 0.99; 95% CI = 0.83 to 1.15 points). Attention emerged as a confounder of the association between rehabilitation and final FMA-UE (b = 5.5; 95% CI = −0.8 to 11.9 points), influencing both rehabilitation and UL response.

**Conclusion:**

Preserved attention in people who have survived stroke might lead to greater UL motor recovery, albeit estimates have high levels of variability. Moreover, the increase in the dose of rehabilitation can lead to 5.5 points improvement on the FMA-UE, a nonsignificant but potentially meaningful finding. The approach described here discloses a new framework for investigating the effect of rehabilitation treatment as a potential driver of recovery.

**Impact:**

Attentional resources could play a key role in UL motor recovery. There is a potential association between amount of UL recovery and dose of rehabilitation delivered, needing further exploration. Preserved attention and rehabilitation dose are candidate predictors of UL motor recovery.

## INTRODUCTION

People who have survived stroke frequently have upper limb (UL) motor impairments.^1^^,^^2^ Previous research has emphasized several specific factors as potential predictors of UL motor recovery.^3^ These factors include maintenance of shoulder abduction and finger extension (SAFE), as well as preserved conduction and anatomical integrity of the corticospinal tract (CST), investigated by motor evoked potentials (MEPs) and fractional anisotropy (FA) measures.^4^^,^^5^ Recent evidence also suggests that a quantitative assessment of the disconnection of white matter tracts is a strong predictor of the deficits after stroke.^6^ In particular, the CST plays a fundamental role in controlling finger extensors and fine hand movements, and it has been widely investigated as a key element implied in prediction of UL motor outcomes.^4^^,^^5^^,^^7^^,^^8^ However, these factors mostly explain spontaneous recovery, as the amount of rehabilitation has never been considered as a factor potentially influencing the association between those predictors and motor recovery. Currently, most prognostic studies have used the Fugl-Meyer Assessment (FMA) for the upper extremity (FMA-UE)^9^ as the clinical endpoint in UL stroke rehabilitation.^10^^,^^11^

Along with motor impairments, people who have survived stroke may also present with cognitive deficits.^1^^,^^2^ While performing movements, the motor system increases the attentional demand depending on the task complexity, as the execution of goal-directed actions requires planning and processing of motor complexity.^12–15^ Indeed, people who have survived stroke require greater attentional resources to perform specific tasks than do people who are healthy. Consequently, attention may be the most critical cognitive function influencing motor recovery after stroke, as commonalities of the underlying mechanisms of motor and cognitive recovery have been unveiled.^16–19^

The leading hypothesis of this study is that UL motor recovery after stroke might be associated with behavioral, neural, and physiological factors, but driven by the total amount of rehabilitation offered. The aim of the present study is to investigate the association between dose of rehabilitation and UL motor outcome after stroke, identifying which features might be potential predictors of rehabilitation-induced motor recovery.

## METHODS

For a full and comprehensive reporting of the present study, the Strengthening the Reporting of Observational Studies in Epidemiology statement has been used.^20^ Full and detailed methods of the technical aspects of this study are contained in our protocol paper.^21^ Here, only clinical (ie, motor, cognitive), and instrumental (ie, transcranial magnetic stimulation [TMS] and magnetic resonance imaging [MRI]) data have been considered, having an already established background in the prognostic framework. The funders played no role in the design, conduct, or reporting of this study.

### Study Design

This was a longitudinal observational study on a cohort of people who survived stroke and were undergoing in-patient rehabilitation during a period of hospitalization. Patients were enrolled between August 2021 and August 2023 at the IRCCS San Camillo Hospital in Venice (Italy) after providing written consent to participate in the study. Ethical approval was granted by the Comitato etico per la Sperimentazione Clinica della Provincia di Venezia e IRCCS San Camillo (Prot. 1375/IRCCS San Camillo). The protocol has been registered on ClinicalTrials.gov (NCT05423119).

### Participants

Study participants were recruited from among people who survived stroke and were admitted for a period of neurorehabilitation treatment. Inclusion criteria were ≥ 18 years old and first-ever supratentorial ischemic or hemorrhagic, unilateral stroke, based on medical records. Patients were excluded in case of bilateral or pure cerebellar lesion; presence of non-stabilized fractures; diagnosis of other neurological and/or psychiatric disorders; unstable medical condition (eg, untreated seizures); any other relevant UL musculoskeletal impairment potentially hampering the assessment; and inability to provide informed consent. Specific exclusion criteria related to the instrumental technology (ie, MRI and TMS) employed in this study will be detailed in each specific section.

### Study Procedures

Each participant underwent clinical (ie, motor and cognitive) assessments and neuroanatomical (ie, MRI) and neurophysiological (ie, TMS) investigations within 10 days from admission and at 8 weeks after admission (or before discharge if <8 weeks). Participation to the study did not result in the exclusion or reduction of the standard treatment for the patients.

### Neuromotor Rehabilitation

Every participant underwent a tailored motor rehabilitation program, consisting of a minimum of 1 h of conventional therapy per day. Whenever appropriate, 1 or more hours of other modalities such as technology-based rehabilitation (eg, robotics, virtual reality) and/or occupational therapy were delivered, according to the individual rehabilitation plan designed by the rehabilitation team. General physical therapy consisted of motor control and task-oriented exercises, focusing on coordination, proprioception, and daily-tasks performance. The amount of motor rehabilitation delivered was retrievable from medical records only at patient discharge. Thus, we did not have the actual number of hours received in 8 weeks, but the total number of hours patient received during the whole stay in the hospital. Indeed, according to the prescriptions of the Italian National Health System, people who have survived stroke are typically hospitalized for 8 weeks of rehabilitation (ie, 40 days of treatment considering that rehabilitation is delivered from Monday to Friday), but it may happen that due to clinical needs patients are hospitalized a little longer or shorter. Therefore, rehabilitation dose was quantified in total hours of neuromotor intervention (ie, physical therapy) received, adjusted for the actual number of days patients underwent rehabilitation, according to the following formula: (total hours received/days of rehabilitation) × 40. This formula allows to normalize hours of rehabilitation received per days in order to avoid comparing absolute number of hours regardless of the length of stay.

Even though UL-specific activity dose was retrievable, the dose of the total rehabilitation was used as suggested by previous evidence highlighting that dose can be more influential than content if delivered with appropriate modalities and intensity.^22–24^

### Behavioral Outcomes for Motor and Cognitive Profiles

The primary outcome of this study was the FMA-UE (0–66 points).^9^ For quantification of stroke severity, the National Institutes of Health Stroke Scale (NIHSS) was used (0–42 points).^25^ Moreover, motor abilities and impairments were described using the Functional Independence Measure (FIM) for autonomy in activities of daily living^26^ (13–126 points), the Medical Research Council muscle strength scale for assessment of voluntary force, applied to SAFE^27^ (0–10 points). Other measures acquired were FMA for sensation function,^9^ Box & Blocks Test,^28^ Trunk Control Test,^29^ and Modified Ashworth Scale (MAS)^30^ at the biceps brachii and flexor carpi.

All patients underwent a comprehensive neuropsychological assessment. Two tests were administered to screen for deficits in general cognitive functioning (Mini-Mental State Examination)^31^ and specific cognitive functions (Oxford Cognitive Scale [OCS]).^32^ In particular, the OCS presents different subtests assessing specific cognitive functions. For the purpose of this study, we considered only attention-related scores retrieved from the OCS. The attention score relates to a visual search task, in which participants are presented with a sheet reporting both whole and broken hearts (eg, heart with a missing piece either on the left or on the right of the figure). The total score of the subtest refers to the number of whole hearts marked correctly in 3 min (maximum score = 50 points). Based on their performance, participants’ attention was classified as impaired or not, according to the standardized cut-off values depending on age and educational levels for the Italian population, as described in the manual.^32^

### Neurophysiological and Neuroanatomical Features: TMS and MRI Outcomes

Neurophysiological (ie, TMS-based) assessment was applied to eligible patients according to the most updated guidelines.^33^ TMS was administered by means of single-pulse stimuli on primary motor cortex representation of the extensor digitorum communis muscle of the damaged hemisphere. More specifically, for the impaired limb, patients were classified as positive MEPs when it was possible to elicit contralateral MEPs, at rest and with increasing levels of stimulation. If this was not possible, we tried to elicit MEPs by asking to the patients to exploit increasing levels of muscular pre-activation (again, if this procedure was successful, patients were classified as MEP positive). If absence of the response persisted, patients were classified as MEP negative (ie, no possibility to individuate contralateral MEPs from impaired limb). Exclusion criteria to TMS procedure included the presence of medical history for seizure or epilepsy, as well as the presence of heart disease or body-inserted devices.

Among patients with valid MRI acquisition, we included patients with distinguishable lesion in FLuid-Attenuated Inversion Recovery (FLAIR) sequence and unilateral hemispheric lesion, thereby excluding images with a bilateral lesion. MRI data were used to derive the following measures: lesion volume, CST disconnection proportion, FA, and Fractional Anisotropy Asymmetry Index (FAAI). Participants with contraindications to MRI scanning (including but not limited to a history of claustrophobia, certain metallic implants, and metallic injury to the eye) were excluded.

For a more detailed description of TMS and MRI acquisition protocols, please see the protocol paper.^21^

### Neuroimaging Data Processing

T1-weighted MRI and diffusion tensor MRI (DTI) images were used for this study. For a detailed description of the processing steps used to extract neuroimaging measures (ie, lesion volume, CST disconnection proportion, FA, FAAI), please see the Supplementary Material. Very briefly, from T1-weighted MRI scans (or computerized tomography when MRI was unavailable), each patient’s lesion was segmented and normalized into the MNI152 space in order to proceed with the extraction of white matter disconnection maps and CST disconnection proportion by means of Brain Connectivity and Behaviour toolkit (BCBtoolkit) software (http://toolkit.bcblab.com).^6^ Note that these measures are derived from brain scans that are usually acquired as part of standard clinical imaging protocols. In addition, for patients who underwent full MRI imaging, FA maps were generated from DTI scans using DTIFit, part of Functional Magnetic Resonance Imaging of the Brain (FMRIB)’s Diffusion Toolbox. FA and FAAI were used as indices for baseline characteristics of patients’ CST integrity.

### Data Analysis

Data were summarized as mean and ±1 standard deviation (SD) or median and interquartile range, as appropriate. Metrics of interest were reported with variation expressed as mean difference with 95% CIs. The Wilcoxon signed rank test or the McNemar test was used to test whether paired means or frequencies were statistically different. For FMA-UE, effect size was calculated with the Cohen *d*.^34^

**Figure 1:**
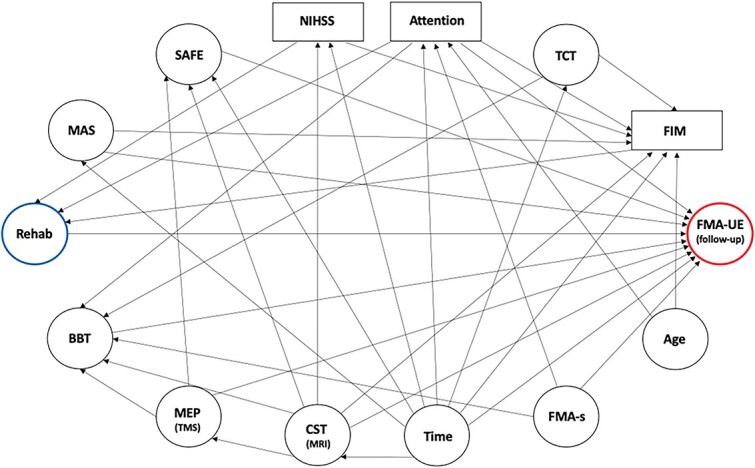
Directed Acyclic Graph (DAG) Representing Variables and Their Relationships. Arrows denote the direction of assumed relationships among selected variables. Note that the baseline Fugl-Meyer Assessment (FMA) for upper extremity (FMA-UE; outcome, circled in red) is not included in the DAG because it was used as a fixed adjustment variable in the model. BBT = box & blocks test; CST = corticospinal tract; FIM = functional independence measure; FMA-s = FMA for sensation; MAS = Modified Ashworth Scale (at biceps brachii); MEP = motor evoked potential; MRI = magnetic resonance imaging; NIHSS = National Institutes of Health Stroke Scale; Rehab = rehabilitation (exposure in hours, blue); SAFE = shoulder abduction and finger extension; TCT = trunk control test; TMS = transcranial magnetic stimulation.

We investigated the association between dose of rehabilitation and FMA-UE, controlling for the baseline FMA-UE, using ordinary least squares (OLS) regression as the primary analysis. We used our knowledge to guide multivariable model building without relying on automatic variable selection strategies. To obtain unbiased estimates of such relationships, adjusting covariates were selected using a directed acyclic graph (DAG).^35^ DAGs provide an intuitive yet rigorous tool in clinical research because they are constructed to depict prior knowledge (and/or explicit hypotheses) about the causal relationships between variables of interest (including the outcome). We adopted this method to perform variable selection, which was used instead of automatic approaches.^36^ Under this framework, we first built relationships among variables usually considered in neurological rehabilitation to inform clinical judgment about the prognosis of people who have survived stroke. Therefore, we determined several pathways defining how these variables can influence both the exposure and/or the outcome (Figure 1).

We assumed that the effects of rehabilitation (the exposure) could be reliably captured by FMA-UE at follow-up (the outcome). Second, we hypothesized that dose of rehabilitation is influenced by the level of stroke severity (NIHSS), attention (OCS), and independence (FIM). Third, we hypothesized that motor recovery (FMA-UE at follow-up) could be influenced by time from lesion, baseline CST integrity, motor function (SAFE, Box & Blocks Test, MAS), sensation function (FMA), and attention (OCS). Time from lesion could also influence NIHSS, motor function (SAFE, FIM, FMA-UE, MAS, Trunk Control Test), neural features (CST MRI), and attention (OCS), as well as age could influence attention (OCS) and independence (FIM). Besides, CST integrity (assessed by TMS and MRI) could influence motor function (SAFE, Box & Blocks Test, FMA-UE). Provided all these relationships, FIM, NIHSS and attention were considered the minimum adjusting covariates to get an unbiased statistical association between the exposure and the outcome.

As sensitivity analyses, we fitted OLS models using TMS, CST, and time from lesion as further adjusting covariates, chosen due to their role in the literature published so far.^4^^,^^5^^,^^7^^,^^8^ To obtain an expected shrinkage factor of 0.9 for a model with four covariates and an anticipated *R*^2^ of 0.6, a sample size of at least 33 patients is required. This would ensure a multiplicative margin of error of ≤1.4, based on an estimated FMA-UE of 37.2 (SD = 23.2).^3^

Multiple imputations using a nonparametric approach in conjunction with bootstrap were used to reduce bias in OLS estimates, due to the extent of missing data in the selected covariates (5% OCS, 25% TMS, and 40% MRI), whose pattern was explored with recursive partitioning and assumed missing at random. The imputation model included all candidate parameters and the endpoint indicator, with all terms expressed as linear function, whose functional forms were graphically checked. Inference on considered parameters was obtained by combining estimates over 10 to 40 imputed datasets using the Rubin rules.^37^ Plausibility of the estimates over complete case analysis was graphically assessed. Multiple imputed data were used throughout all model fitting and evaluation steps. Considering an event per variable of <10, estimates of the primary analysis were reported also applying a shrinkage factor.^38^

Our choice of which outcome measures/variables to be included in the protocol design and in the statistical data modeling was driven by several considerations. Regarding the protocol design, we selected outcome measures recommended by the core outcome set for stroke rehabilitation^10^^,^^39–41^; outcome measures with the highest predictive value^4^^,^^5^^,^^8^^,^^15^; and a total number of outcome measures granting a careful patient’s evaluation, but at the same time keeping the total duration feasible for the patient (ie, avoiding a high rate of dropouts due to patient’s fatigue during the evaluation). For the statistical modeling, we selected the variables of interest in order to obtain unbiased estimates and valid confidence intervals of the association between exposure and outcome.

All models were validated and calibrated using 500 bootstraps; overall performance and predictive ability were reported as mean squared error (MSE), mean absolute difference (MAD), optimism-corrected *R*^2^ index, and the heuristic shrinkage estimate of van Houwelingen and Le Cessie.^42^ Model estimates are accompanied with 95% CIs. All analyses were performed using R Core Team,^43^ version 4.3.0, with rms, Hmisc, and dagitty packages added.

### Role of the Funding Source

The funders provided support in the form of salaries for the authors or covered expenses required for the project, but did not have any additional role in the study design, data collection and analysis, decision to publish, or preparation of the manuscript.

## RESULTS

**Figure 2:**
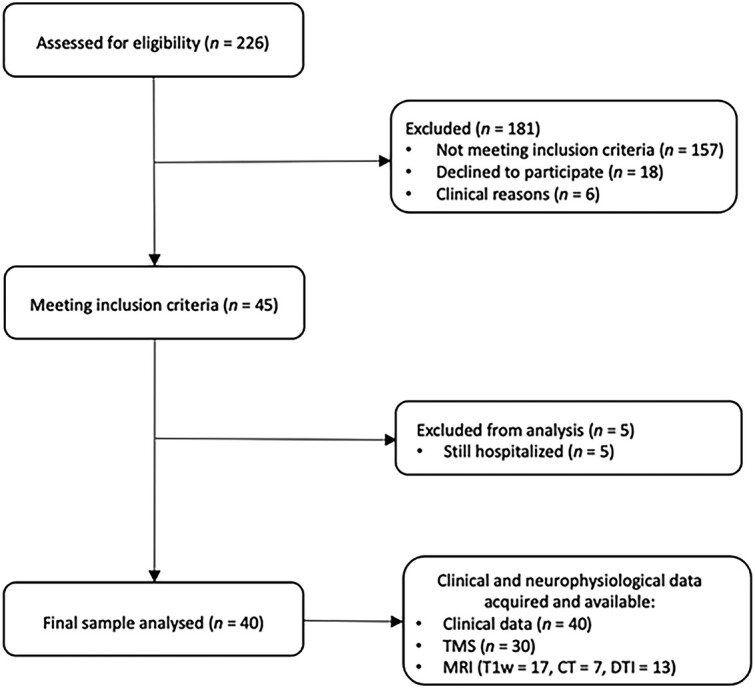
Flowchart of the Study Population. CT = computerized tomography; DTI = diffusion tensor magnetic resonance imaging (MRI); T1w = T1 weighted; TMS = transcranial magnetic stimulation.

Among 226 people who survived stroke and were assessed for eligibility, 40 of them, between 43 and 84 years old, were included in the analyses. A comprehensive flow-chart of the study is presented in Figure 2, whereas Table 1 reports the main characteristics of participants at baseline.

**Table 1:** Overview of Sample Characteristics at Baseline and Rehabilitation Dose (N = 40)*^a^*

**Sample Characteristics**	**Value**
Sociodemographic characteristics	
Sex, patient reported as male/female	24 (60)/16 (40)
Age, y, mean (SD)	65.8 (11.68)
Type of stroke, ischemic/hemorrhagic	23 (58)/17 (42)
Hemisphere affected, right/left	21 (53)/19 (47)
Dominant side affected, yes	14 (35)
Time from lesion, mo, median (IQR)	3.8 (1.2–3.8)
NIHSS, mean (SD)	7.18 (4.29)
Rehabilitation dose, mean (SD)	
Days of rehabilitation	48.65 (23.6)
Total dose of upper limb rehabilitation, h	27.3 (20.7)
Total dose of rehabilitation, h	58.2 (22.8)
Total rehabilitation/per day, min	87.3 (34.2)
Neurophysiological and neuroanatomical characteristics	
MEP, positive/negative, n = 30	18 (60)/12 (40)
FA PLIC, n = 13, mean (SD)	0.6 (0.06)
FAAI PLIC, mean (SD)	0.07 (0.06)
CST disconnection proportion, n = 24, mean (SD)	0.14 (0.15)
Volume of CST disconnection, mean (SD)	22,956.68 (17,802.71)

^a^ Values are reported as numbers (percentages) unless otherwise indicated. CST = corticospinal tract; FA = fractional anisotropy; FAAI = Fractional Anisotropy Asymmetry Index; IQR = interquartile range; MEP = motor evoked potential; NIHSS = National Institutes of Health Stroke Scale; PLIC = posterior limb internal capsule.

Table 1 also reports information about type and dose of rehabilitation. On average, patients were administered 49 days of rehabilitation, with a minimum of 15 and a maximum of 136 days, corresponding to 1.5 h of rehabilitation per day, almost half of the time with UL-specific activities. Some patients (40%; 16/40) also received technology-based rehabilitation.

Among the overall sample of 40 patients, 30 (75%) of them underwent TMS (four declined the examination and six had contraindications) and therefore had MEP assessments (Table 1). For the analyses of MRI data (Table 1), two patients were excluded in accordance with imaging inclusion criteria, 17 declined to take part in the MRI scan, and four had contraindications, for a final sample of 17 patients who underwent the MRI protocol. For all of them, T1-weighted images were available and usable, but only DTI data from 13 patients survived the quality check (data from four were excluded due to excessive motion). For seven patients who did not have MRI scans, we used previously acquired computerized tomography scans. Therefore, for 24 patients we could extract the tract disconnection proportion. Based on our hypothesis, we limited DTI data extraction to CST. More than half of the patients were MEP positive and had, on average, 14% CST disconnection.

During the study period, FMA-UE increased, on average, by 4.7 points (*P <* .001), as well as other clinical variables showing a positive change, as reported in Table 2. MAS, FMA for sensation function, and OCS scores were the only variables which did not show any evidence of statistical or clinical change. In particular, FMA-UE improved with a moderate effect size (Cohen *d* = 0.55).

**Table 2:** Modifications of Behavioral Outcome Measures After Rehabilitation*^a^*

**Outcome Measure**	**Baseline (N = 40)**	**Follow-up (N = 40)**	***P***	**Difference** ^***b***^ **(95% CI)**
FMA for upper extremity	27.9 (23.4)	32.5 (25.0)	<.001	4.7 (1.9 to 7.5)
FMA for sensation function	17.8 (6.7)	17.6 (7.2)	.903	0.0 (−1.7 to 1.6)
BBT	10.3 (16.7)	16.2 (21.2)	<.001	5.8 (2.4 to 9.2)
TCT	69.9 (27.3)	81.6 (25.2)	.007	10.9 (3.6 to 18.2)
MAS at the biceps brachii	1.0 (0.0–1.0)*^c^*	1.0 (0.0–1.0)*^c^*	.829	0.0 (−1 to 1)*^d^*
MAS at the flexor carpi	1.0 (0.0–1.3)*^c^*	1.0 (0.0–1.5)*^c^*	.644	0.0 (−0.5 to 1.0)*^d^*
FIM	85.1 (23.4)	96.2 (21.3)	<.001	10.6 (6.6 to 14.5)
SAFE	4.6 (3.5)	5.3 (3.5)	.007	0.6 (0.2 to 0.9)
OCS for attention, impaired	24 (60)*^e^*	19 (48)*^e^*	.683*^f^*	−12.5 (−36.7 to 11.7)*^g^*

^a^ Values are reported as mean and ± 1 SD unless otherwise indicated. BBT = box & blocks test; FIM = functional independence measure; FMA = Fugl-Meyer Assessment; MAS = Modified Ashworth Scale; OCS = Oxford Cognitive Scale; SAFE = shoulder abduction and finger extension; TCT = trunk control test.

^b^ Mean difference unless otherwise indicated.

^c^ Reported as median (quartile 1–quartile 3).

^d^ Median difference.

^e^ Reported as numbers (percentages).

^f^ McNemar test.

^g^ Proportional difference.

**Figure 3:**
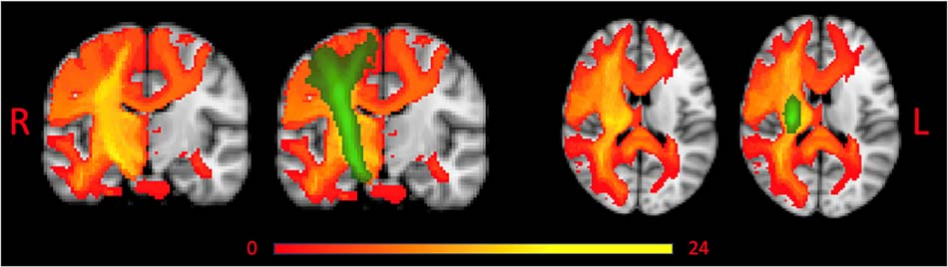
Overlap of Tract Disconnection Across Patients Without and With Corticospinal Tract (CST) Overlay. Red to yellow represents tract disconnection across patients. Green represents the CST. L = left; R = right.

Overlap of tract disconnection across participants is shown in Figure 3.

### Descriptive Multivariable Modeling

The OLS regression model with FIM, NIHSS, and OCS for attention as adjusting covariates and values of FMA-UE at baseline as independent variables was statistically significant (*F* = 90.2; *P <* .001). No evidence of association was found between FIM, NIHSS, OCS for attention, dose of rehabilitation, and final FMA-UE (Table 3). From the fitted model, increasing rehabilitation from 40 to 60 h resulted in an average increase of 1.4 (95% CI = −1.3 to 4.0; *P* = .2992) points at the FMA-UE. This estimate became larger (2.7; 95% CI = −2.5 to 8.0 points) when rehabilitation increased by 40 h,

**Table 3:** Fitted Model After Multiple Imputation*^a^*

**Model Parameters**	**Estimate**	**SE**	**Shrunken Estimate**	**95% CI for Estimates**
Intercept	16.00	10.15	15.75	−4.6 to 36.62
FMA for upper extremity at baseline	0.99	0.08	0.97	0.83 to 1.15
Total dose of rehabilitation (h)	0.07	0.07	0.07	−0.06 to 0.20
FIM	−0.10	0.08	−0.10	−0.25 to 0.06
NIHSS	−0.42	0.48	−0.41	−1.40 to 0.56
Level of attention (impaired vs normal)	−5.55	3.14	−5.46	−11.93 to 0.83

^a^ FIM = functional independence measure; FMA = Fugl-Meyer Assessment; NIHSS = National Institutes of Health Stroke Scale; SE = Standard Error.

up to 5.5 (95% CI = −5.1 to 16.0) points of improvement when rehabilitation was delivered for 120 h. In accordance with the DAG, attention confounded the association between rehabilitation dose and FMA-UE, potentially influencing both the exposure and the outcome. Numerically, attention was a negative confounder, since it underestimated the association between rehabilitation dose and UL recovery by 50%. In particular, FMA-UE at follow-up was expected to be 5.5 (95% CI = −0.8 to 11.9) points higher in patients with nonimpaired attention than in patients with impaired attention (*P =* .0862).

In our model, FMA-UE at baseline remained the only statistically significant factor associated with UL recovery, explaining 54.4% of the FMA-UE variation.

Overall, the optimism-corrected *R*^2^ of this model is 0.862, with a shrinkage factor of 0.985. Considering an MSE of 84.26, although not meant for prediction, this model will validate on new data ~5.5% worse than on this dataset, with an average absolute difference between the FMA-UE observed values and the predicted values of 2.3 points.

The fitted model for the sensitivity analyses with added information derived from TMS (*R*^2^ = 0.856; MSE = 88.6; MAD = 1.5; rehabilitation coefficient = 0.06; 95% CI = −0.08 to 0.19) or MRI (*R*^2^ = 0.851; MSE = 91.5; MAD = 2.2; b for rehabilitation = 0.07; 95% CI = −0.07 to 0.21) or using the time from lesion as a further adjusting covariate (*R*^2^ = 0.846; MSE = 95.2; MAD = 2.7; b for rehabilitation = 0.06; 95% CI = −0.08 to 0.20) performed worse than the primary analysis, with <1% of the variation of FMA-UE explained by one of each of these factors. The magnitude of the association between rehabilitation dose and FMA-UE did not show clinically significant variation compared to estimates presented in the primary analysis.

## DISCUSSION

Here we have shown that preserved attention in people who have survived stroke might lead to greater UL motor recovery, albeit estimates have high levels of variability. Moreover, an increase in as the dose of rehabilitation can lead to 5.5 points improvement on the FMA-UE, which suggests a potential clinically significant association between higher dose of rehabilitation and better UL motor function recovery. Despite the results are not statistically significant, the clinical relevance of our findings if replicated in other cohorts, can be substantial. Indeed, our observation, makes preserved attention and the dose of rehabilitation potential candidate predictors of motor recovery.^44^

Guidelines state that at least 3 h/day of rehabilitation should be delivered in subacute and chronic phase.^45^ Furthermore, studies delivering up to 90 to 300 h, in 3 to 12 weeks (ie, 5–6 h/d), have shown a change of 9 to 11 points at the FMA-UE, representing a significant clinical impact.^18^^,^^23^^,^^24^ In our study, despite patients performed half of the recommended dose (90 min/d), their mean change was nearly 5 points, which is still considered clinically relevant.^46^ This finding implies that our treatment dose may not have been as extensive as in studies administering a substantial amount of therapy, yet it was adequate to yield noteworthy motor improvement from a clinical perspective.

Current evidence on the effect of time since stroke onset on UL recovery, was observed from hyperacute (3 days) to early subacute phases (3 months), when time-dependent spontaneous recovery is the driving factor.^47^ Conversely, our population is representative of the late subacute phase (>3 months after stroke) and time from lesion onset was not a significant factor associated with UL recovery. Most importantly, time from lesion did not modify the magnitude of dose of rehabilitation when added as further adjusting factor. Moreover, we did not find any evidence supporting the relevance of neurophysiological and brain structural information when explaining the association between rehabilitation dose and UL response. Indeed, sensitivity analyses with TMS and MRI did not explain more variability than primary analysis alone, and coefficients of dose of rehabilitation did not change at all after including neurophysiological and neuroanatomical information into the models (despite CST disconnections were overlaid in all our patients), leaving to the integrity of the CST a small role in explaining the FMA-UE variability. On the other hand, other studies found structural (eg, FAAI) and functional (MEP-positive) integrity of the CST as a strong predictor of recovery. However, this evidence considered only UL spontaneous recovery.^4^^,^^5^ Concurrently, based on FIM and NIHSS coefficients, patients with low level of independence but less severe neurological status may have greater motor improvement than those with higher levels of independence but worse neurological severity.

As suggested by other evidence, we also explored the influence of attentional function and white matter disconnections on motor improvement.^6^ Our results clearly indicate that attention change the magnitude of estimates, thus confounding the association between rehabilitation dose and UL recovery. This result supports the hypothesis that attentive function sustains motor recovery, throughout shared large-scale brain networks, connecting both cognitive and motor areas.^15^ Furthermore, cognitive impairments might change responsiveness to motor rehabilitation, affecting the final outcome of targeted interventions after stroke.^48^

### Study Limitations

The present study relies on authors’ assumptions about different relationship between clinical, neurophysiological, and neural features used in usual subacute-chronic stroke rehabilitation setting. These were used to realize the DAG, which was implemented as a means for variable selection only. Therefore, no conclusion must be drawn about the causal relationship between each of the factors considered. Moreover, our sample size limited the number of variables to be fit in the sensitivity analysis and power, consequently. Besides, we assumed that FMA-UE could reliably detect rehabilitation effects. The FMA-UE scale has an important ceiling effect, which limits its ability to detect changes over time.^49^ Furthermore, as shown in the primary analysis, baseline FMA-UE explained 54% of FMA-UE variation, and it was the only factor statistically associated with the outcome. This means that the final value is strongly dependent from the initial value, and probably they are not even linearly related, which may challenge the interpretation of any association. However, compared to literature using absolute or percentage change of FMA-UE as primary endpoint, we properly implemented analysis of covariance as a more powerful statistical approach.^15^ Other metrics and modeling strategies should be explored to assess the effect of rehabilitation, considering that measures of effects should be as independent of baseline values as possible.

## CONCLUSION

This is the first study investigating the association between multimodal factors and dose of rehabilitation delivered to people who have survived stroke. From our perspective, this approach discloses a new framework for investigating the effect of rehabilitation treatment as a potential driver of recovery. The dose of rehabilitation used in our study is not sufficient to capture a significant UL motor recovery change. However, our findings suggest that the level of attention in people who have survived stroke and are undergoing motor rehabilitation could be a crucial element to be considered. This implies for future studies a careful estimation of the level of preserved attention in order to provide an unbiased association of the clinical effect of rehabilitation and the amount of intervention (dosage) with UL motor function recovery.

## Supplementary Material

2023-0811_R2_Supplementary_Materials_au_cjt2_pzae148

## Data Availability

Data can be accessed upon request to the IRCCS San Camillo Hospital according to GDPR and Italian regulations for the privacy of biomedical data. A submission to the local ethical committee and informed consent from the participants may be required.

## CLINICAL TRIAL REGISTRATION

The protocol has been registered on ClinicalTrials.gov (NCT05423119).
