# Supplementary material for: Modeling Upper Limb Rehabilitation-Induced Recovery After Stroke: The Role of Attention as a Clinical Confounder
Source: Phys Ther. 2024 Oct 10;105(2):pzae148. doi: 10.1093/ptj/pzae148 (PMC11878758; doi:10.1093/ptj/pzae148)
Supplement: 2023-0811_R2_Supplementary_Materials_au_cjt2_pzae148 [file 2023-0811_r2_supplementary_materials_au_cjt2_pzae148.pdf]

Supplementary Materials

Neuroimaging data processing

T1w and CT images

Automated brain lesions segmentation was carried out using the Lesion-Identification with-Neighbourhood-Data-Analysis (LINDA) software[1]. The resulting lesion mask (in native space) was visually inspected and manually corrected using the ITK-SNAP software independently by two trained researchers (SS and DD)[2]. Finally, to allow direct comparisons across patients, each lesion was registered to the MNI template using the pipeline of the Brain Connectivity and Behaviour toolkit (BCBtoolkit) software (http://toolkit.bcblab.com), which uses diffeomorphic deformations for image registration[3]. In the case of patients with no available MRI data (e.g., patients refusing scanning or with contraindications to MRI), the lesion was manually segmented from Computerized Tomography (CT) scans (acquired on hospital admission) and then normalized into MNI152-space using RegLSM software in Matlab[4].

After normalization, the disconnection maps were estimated by means of BCBToolkit software, in which each MNI-registered lesion map was used as a seed to track probable passing tracts using 176 healthy controls from the “Human Connectome Project” 7T diffusion-weighted dataset. Lastly, the disconnection proportion for all tracts was computed through the software Tractoctron in the BCBtoolkit [3], from which CST disconnection proportion was extracted. CST disconnection proportion was computed in terms of number of damaged voxels out of the total number of voxels defining the specific WM tract[3]. We also extracted the volume of CST disconnection maps (i.e. damaged voxels, in mm^3) using FSL tools. To overcome the problem of small sample size and compare all patients, these data were considered in relation to damaged and non-damaged hemisphere. For visualisation

purposes, lesioned brains were flipped to all be on the same hemisphere. In details, disconnection maps from patients with left hemisphere strokes were flipped about the midline after registration to standard space so that all maps appeared on the right side (Figure 3).

DTI data

As DTI is particularly sensitive to susceptibility-induced distortions, we have adopted a correction strategy based on the complementary information from pairs of diffusion images acquired with reversed phase-encoding (PE) directions to correct for distortions[5]. Subsequently, FA maps for each participant were generated using DTIFit, part of FMRIB's Diffusion Toolbox, that fits a diffusion tensor model at each voxel. FA and FAAI were used to index for baseline characteristics of patients' CST integrity. In detail, FA values range between 0 (low anisotropy, i.e. reduced WM integrity) and 1 (high anisotropy). FAAI has been calculated as $(FA_{unaffected}–FA_{affected})/(FA_{unaffected}+FA_{affected})$, the values range between -1 and + 1 where positive values indicate lateralisation towards the unaffected side, whereas FAAI=0 suggest symmetric FA between the hemispheres[6, 7].

References

1. Pustina D, Coslett HB, Turkeltaub PE, et al. Automated segmentation of chronic stroke lesions using LINDA: Lesion identification with neighborhood data analysis. *Hum Brain Mapp* 2016; 37: 1405-1421. 2016/01/13. DOI: 10.1002/hbm.23110.
2. Yushkevich PA, Pashchinskiy A, Oguz I, et al. User-Guided Segmentation of Multi-modality Medical Imaging Datasets with ITK-SNAP. *Neuroinformatics* 2019; 17: 83-102. 2018/06/28. DOI: 10.1007/s12021-018-9385-x.
3. Foulon C, Cerliani L, Kinkingnehun S, et al. Advanced lesion symptom mapping analyses and implementation as BCBtoolkit. *Gigascience* 2018; 7: 1-17. 2018/02/13. DOI: 10.1093/gigascience/giy004.
4. Biesbroek JM, Kuijf HJ, Weaver NA, et al. Brain Infarct Segmentation and Registration on MRI or CT for Lesion-symptom Mapping. *J Vis Exp* 2019 2019/10/15. DOI: 10.3791/59653.
5. Andersson JLR, Graham MS, Drobnjak I, et al. Towards a comprehensive framework for movement and distortion correction of diffusion MR images: Within volume movement. *Neuroimage* 2017; 152: 450-466. 2017/03/13. DOI: 10.1016/j.neuroimage.2017.02.085.

6. Stinear CM, Barber PA, Petoe M, et al. The PREP algorithm predicts potential for upper limb recovery after stroke. *Brain* 2012; 135: 2527-2535. 2012/06/13. DOI: 10.1093/brain/aws146.
7. Stinear CM, Byblow WD, Ackerley SJ, et al. PREP2: A biomarker-based algorithm for predicting upper limb function after stroke. *Ann Clin Transl Neurol* 2017; 4: 811-820. 2017/11/22. DOI: 10.1002/acn3.488.
